# C^2^DAN: An Improved Deep Adaptation Network with Domain Confusion and Classifier Adaptation

**DOI:** 10.3390/s20123606

**Published:** 2020-06-26

**Authors:** Han Sun, Xinyi Chen, Ling Wang, Dong Liang, Ningzhong Liu, Huiyu Zhou

**Affiliations:** 1 College of Computer Science and Technology, Nanjing University of Aeronautics and Astronautics, Nanjing 211106, China; 17817356323@163.com (X.C.); wl941017@163.com (L.W.); liangdong@nuaa.edu.cn (D.L.); liunz@163.com (N.L.); 2 MIIT Key Laboratory of Pattern Analysis and Machine Intelligence, Nanjing 211106, China; 3 School of Informatics, University of Leicester, Leicester LE1 7RH, UK; hz143@leicester.ac.uk

**Keywords:** transfer learning, domain adaptation, MK-MMD, domain confusion, classifier adaptation, vehicle classification

## Abstract

Deep neural networks have been successfully applied in domain adaptation which uses the labeled data of source domain to supplement useful information for target domain. Deep Adaptation Network (DAN) is one of these efficient frameworks, it utilizes Multi-Kernel Maximum Mean Discrepancy (MK-MMD) to align the feature distribution in a reproducing kernel Hilbert space. However, DAN does not perform very well in feature level transfer, and the assumption that source and target domain share classifiers is too strict in different adaptation scenarios. In this paper, we further improve the adaptability of DAN by incorporating Domain Confusion (DC) and Classifier Adaptation (CA). To achieve this, we propose a novel domain adaptation method named C^2^DAN. Our approach first enables Domain Confusion (DC) by using a domain discriminator for adversarial training. For Classifier Adaptation (CA), a residual block is added to the source domain classifier in order to learn the difference between source classifier and target classifier. Beyond validating our framework on the standard domain adaptation dataset office-31, we also introduce and evaluate on the Comprehensive Cars (CompCars) dataset, and the experiment results demonstrate the effectiveness of the proposed framework C^2^DAN.

## 1. Introduction

In recent years, deep learning has made great achievements in a large number of computer vision tasks, such as image recognition [1,2,3], object detection [4,5], fine-grained classification [6,7], semantic segmentation [8,9] and so on. For such satisfactory results, training with huge labeled datasets is essential, but in real applications, data labeling is too expensive, and sometimes impracticable. For example, in video surveillance, images can be affected seriously by camera position and illumination variance, and the application scenarios are also ever-changing, so these factors make the task of labeling the entire dataset impossible. In order to solve such problems, Domain Adaptation (DA) [10] is a very efficient method, which aims to extend the prediction model learned from the source domain with abundant labeled data to the target domain which only has the unlabeled or a small number of labeled data. Here the data between the source and target domain are different but related. 

The main idea of domain adaptation is to reduce the difference between domains and to learn a prediction model. Two types of mechanism are used most to address domain shift: discrepancy-based and adversarial-based. The representative discrepancy-based methods [11,12,13,14,15,16,17,18,19,20] minimize the domain discrepancy by using a distance metric such as Maximum Mean Discrepancy (MMD) [17], CORAL [18,19] and Kullback-Leibler divergence [20], among which MMD-based methods are widely used. In this sort of methods, the difference between source and target domains is usually reduced by optimizing the MMD in Reproducing Kernel Hilbert Space (RKHS), and the feature representation with domain invariance needs to be learned. Adversarial-based methods [21,22,23,24,25,26,27,28,29,30] use a discriminator to distinguish whether the data comes from the source domain or the target domain, so as to increase the domain confusion and minimize the distance between the source and the target domain distribution.

However, these methods are not without problems. The typical discrepancy-based Deep Adaptation Network (DAN) framework [12] which is superior to other similar methods still faces performance degradation problems in feature level transfer. Since adversarial-based methods show efficiency in DA, we investigate herein if combining a discrepancy-based method with adversarial learning will be helpful to improve the performance. In addition, most previous works are based on the assumption that the source domain and target domain share the classifier, but the assumption is too strict to maintain stable effects in different adaptation scenarios, so it is necessary to relax the assumption and reduce the differences between source classifier and target classifier. 

In this paper, inspired by the adversarial-based method [21] and Domain Adaptation with Residual Transfer Networks (RTN) [14], we propose our framework called C^2^DAN which improves the DAN framework by combing it with Domain Confusion (DC) and Classifier Adaptation (CA). These changes make the extracted feature representation more adaptive to the target domain. At the same time, aiming at solving the problem of different adaptation effects of MK-MMD in different scenarios, this paper explores the suitable weight selection of MK-MMD. In addition, we pursue the best combination of MK-MMD, DC and CA to further enhance the efficiency of domain adaptation. Experiments on the standard domain adaptation dataset Office-31 [31] and the CompCars [32] dataset which is used for vehicle classification task demonstrate the effectiveness of our proposed method C^2^DAN. 

The contributions of this paper are as follows:

(1) We combine Domain Confusion (DC) with MK-MMD in DAN for both feature alignment and domain alignment, which makes the model more generalized in the target domain.
(2) The model is extended by adding Classifier Adaptation (CA) to minimize the difference of source classifier and target classifier, the accuracy of the proposed method is further improved.
(3) The best combination of MK-MMD, DC and CA in different scenarios is obtained through experiments on office-31 and CompCars dataset, the experimental results show that our improved method C^2^DAN surpass the performance of DAN.

The structure of this paper is as follows: Section 1 briefly introduces the research objectives and contributions of this paper. Section 2 gives a review of related works about deep domain adaptation. In Section 3, the principle and implementation of the deep fusion method between MK-MMD, DC and CA are proposed. In Section 4 the experimental results on dataset Office-31 are demonstrated and analyzed. The suitable weight selection of MK-MMD in different scenarios is explored. The best combination of MK-MMD, DC and CA is also discussed in Section 4. The details and results of our vehicle classification experiments are shown in Section 5. The conclusions of this paper are summarized in Section 6. 

## 2. Related Work

For the deep domain adaptation methods, the basic criterion is to add the adaptation metric after selecting the adaptive layer, and then to fine tune the network. At the PRICAL meeting in 2014, the Domain Adaptive Neural Network (DaNN) concept [11] was put forward. This network has only two layers of neurons, which include a feature layer and a classifier layer. The characteristic of this network lies in the MMD adaptive layer after feature layer, which calculates the distance in Reproducing kernel Hilbert space (RKHS) between the source domain and target domain, and then optimizes its loss, but because of the poor ability of feature representation based on the shallow network, it is also difficult to solve practical problems. After this, many researchers combined this idea into deep networks. These works adopt AlexNet trained on ImageNet for domain adaptation. The main idea is to fix the first seven layers and add MMD metrics before the classifier layer to implement domain adaptation. However, the effect of such single adaptive layer is limited, so the DAN network was proposed in [12]. It uses MK-MMD, which has stronger representation ability by combing the multi-kernel concept. At the same time, three adaptive layers are added into the network, which achieves better classification effect without increasing the training time. At the ICML conference in 2017, JAN [13] was proposed to extend the data adaptive method to the category adaptation, which used Joint MMD (JMMD) metrics. Inspired by the deep residual network (ResNet) [2] framework, RTN [14] learns adaptive classifiers and transferable features from labeled source domains and unlabeled target domains by embedding the adaptation process of classifiers and features into a unified deep network architecture.

Adversarial-based models use a generator to align the source and target domain data in feature space by working against the discriminator. Generative Adversarial Network (GAN) [33] has been transferred into different application scenarios since it was first presented in 2014. Isola et al. proposed [34] and tried to do image translation with conditional GAN (CGAN) [35], allowing the network to learn image to image mapping functions without having to customize features manually. Coupled Generative Adversarial Networks (CoGAN) [36] learns the joint distribution of source domain and target domain data by imposing a constraint on both networks to share parameters, the ability of the network is limited so that the data generated from noise is about the same for both networks. CoGAN consists of two GAN, which is the same as CycleGAN [37], DualGAN [38] and DiscoGAN [39]. Shen et al. proposed the WGDRL [40] metric which is based on WGAN [41] to measure the distance between source domain and target domain. 

In this work, the DAN [12] method is further improved by domain alignment (DC) and classifier alignment (CA). For domain alignment, DC is used to further utilize the source domain information, which makes the extracted features more powerful in the target domain representation. Then CA is added to improves the domain adaptation effect by aligning the classifiers of source domain and target domain. Meanwhile, we explore the applicable weights of MK-MMD under different scenarios and the best combination of the three aspects. We prove the effectiveness of the proposed method both on standard dataset and vehicle classification task.

## 3. C^2^DAN: Improved Deep Adaptive Network

**Figure 1:**
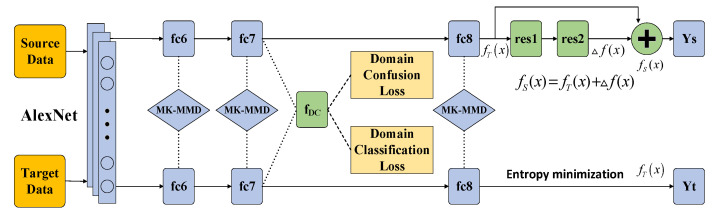
The structure of proposed network C^2^DAN.

The network structure proposed in this paper is based on DAN [12] which adopts multi-layer and multi-kernel MMD. We add a domain classification layer $f_{\mathrm{DC}}$ to perform domain confusion. Domain alignment will be completed when the domain classifier cannot distinguish whether the input is from the source domain or from the target domain. And in order to learn the difference between source classifier and target classifier, a residual block which connects the classifiers of two domain is added to the source classifier. The joint optimization of the four loss functions which include the loss of multi-layer MK-MMD, the loss of $f_{\mathrm{DC}}$ layer, the loss of CA and the final classification loss of the overall network, is used to achieve good unsupervised domain adaptation effect. The network structure of C^2^DAN is shown in Figure 1.

### 3.1. MK-MMD

Multi-kernel Maximum Mean Discrepancy (MK-MMD) is an extension of MMD. MMD is one of the most commonly used non-parametric methods to measure the distribution difference between two domain datasets. The detailed operation is to map the feature representation of source domain and target domain to the reproducing kernel Hilbert space (RKHS), and then calculate the mean distance between the two datasets in RKHS. Given the data distribution s and t of the two domains, and by using the function ϕ(·), the MMD between *s* and *t* is calculated as follows:

(1) $${\mathrm{MMD}}^{2}\left(s,t\right)=\sup_{{\left\|\phi \right\|}_{ℋ}\leq 1}{\left\|E_{x^{s}∼s}\left[\phi \left(x^{s}\right)\right]-E_{x^{t}∼t}\left[\phi \left(x^{t}\right)\right]\right\|}_{ℋ}^{2}$$

where $E_{x^{s}\sim s}\left[\cdot \right]$ represents the mathematical expectation of the source domain’s distribution. And ‖ϕ‖≤1 represents a series of functions in the unit ball of reproducing kernel Hilbert space H. Let $D_{s}={\left\{X_{i}^{s}\right\}}_{i=1}^{M}$ represent sample sets of distribution *s*, then an empirical estimate of MMD can be expressed as follows:

(2) $${\mathrm{MMD}}^{2}\left(D_{s},D_{t}\right)={\left\|\frac{1}{M}\sum_{i=1}^{M}\phi \left(x_{i}^{s}\right)-\frac{1}{N}\sum_{j=1}^{N}\phi \left(x_{j}^{t}\right)\right\|}_{ℋ}^{2}$$

where ϕ(·) represents the feature mapping about $k\left(x^{s},x^{t}\right)=\langle \phi \left(x^{s}\right),\phi \left(x^{t}\right)\rangle$, and $k\left(x^{s},x^{t}\right)$ is usually defined as a convex combination of *L* basis kernels $k_{l}\left(x^{s},x^{t}\right)$, like the following:

(3) $$k\left(x^{s},x^{t}\right)=\sum_{l=1}^{L}\beta_{l}k_{l}\left(x^{s},x^{t}\right),s.t.\beta_{l}\geq 0,\sum_{l=1}^{L}\beta_{l}=1$$

The MMD method is based on single kernel transformation. The multi-kernel MMD (MK-MMD) assumes that the optimal kernel can be obtained by linear combination of multiple kernels. One of the most successful ways to use MK-MMD is DAN. As shown in [12], let *H_k_* denote the RKHS with the characteristic kernel *k*, and let the mean value of distribution *p* in *H_k_* be an independent element $\mu_{k}\left(p\right)$, then we can get $E_{x\sim p}f\left(x\right)=\langle f\left(x\right),\mu_{k}{\left(p\right)\rangle }_{H_{k}}$, where $f\in \left(H_{k}\right)$, The distance of mean value between probability distribution *p* and *q* is expressed as $d_{k}\left(p,q\right)$, and its square formula is as the following:

(4) $$d_{k}^{2}\left(p,q\right)≜{\left\|E_{p}\left[\phi \left(x^{s}\right)\right]-E_{q}\left[\phi \left(x^{t}\right)\right]\right\|}_{{ℋ}_{k}}^{2}$$

Similar to MMD, feature mapping *ϕ* is related to characteristic kernel, so $k\left(x^{s},x^{t}\right)=\langle \phi \left(x^{s}\right),\phi (x^{t})\rangle$. Here $k\left(x^{s},x^{t}\right)$ is defined as a convex combination of *m* PSD kernels $\left\{k_{u}\right\}$. Multi-kernel *k* can use different kernels to enhance the effect of MK-MMD and achieve an optimal and reasonable kernel selection:

(5) $$K≜\left\{k=\sum_{u=1}^{m}\beta_{u}k_{u}:\sum_{u=1}^{m}\beta_{u}=1,\beta_{u}\geq 0,\forall u\right\}$$

*k* is weighted by different kernel and the coefficients $\left\{\beta_{u}\right\}$ is the weight to ensure that the generated multi-kernel *k* is characteristic.

### 3.2. Domain Confusion

**Figure 2:**
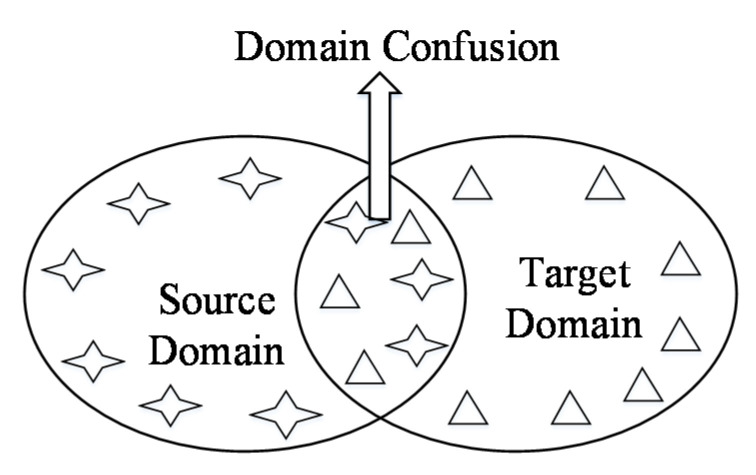
The definition of domain confusion.

In order to reduce the difference of marginal distribution between two domains, maximizing domain confusion (DC) is an effective method. The schematic diagram of domain confusion is shown in Figure 2. For maximizing domain confusion, a domain classification layer $f_{\mathrm{DC}}$ is considered in this paper. The main function of this layer is to judge whether the sample belongs to the source domain or target domain by using the feature representation gained from trained samples. From an intuitive point of view, the more domain-specific the extracted features belong to, the better the effect of domain classification is. Meanwhile the more common the extracted features are, the better the effect of domain confusion is. When the classifier trained by a specific feature representation cannot distinguish the samples of source domain or target domain, we can call this feature representation is domain invariant. In this paper, we use a domain confusion loss, which is optimized to obtain a domain invariant feature representation.

According to [12], for a feature representation $\phi_{repr}$, we measure its domain invariance by learning the best domain classifier based on this representation. $\phi_{D}$ is the parameter of the domain classifier to be learned. By optimizing the following loss function (6), the best domain classifier can be learned. Besides, we introduce the loss of domain confusion for a domain classifier, which calculates cross entropy between the predicted output domain label and a uniform distribution of domain labels, so as to maximize the confusion and reduce the distribution differences between the two domains. The uniform distribution of domain labels means that the possibility of feature representation belonging to source or target domain is $\frac{1}{D}\;$, in which case the domain label is most difficult to judge. D is the number of domains and here D is 2. And the loss function of domain confusion is shown in Equation (7):

(6) $$L_{D}\geq \left(\phi_{repr};\theta_{D}\right)=-\sum_{d}1\left[y_{D}=d\right]\log q_{d}$$

(7) $$L_{conf}\left(\theta_{D};\theta_{repr}\right)=-\sum_{d}\frac{1}{D}\log q_{d}$$

where $y_{D}$ denotes the domain to which the sample belongs, and *q* represents the softmax value of the domain classifier, $q=softmax\left(\theta_{D}f\left(x;\theta_{repr}\right)\right)$.

The optimization of the domain confusion loss above is to find a domain invariant feature representation, in which the best domain classifier cannot achieve good results. Ideally, we want to optimize these two losses simultaneously in the training process. But there are two contradictions between domain classification and domain confusion. Learning a good domain classifier means that the effect of domain confusion is very poor, and also reaching a good domain confusion effect means that the effect of domain classification is very poor. Therefore, the two losses of layer $f_{\mathrm{DC}}$ need to be optimized jointly to achieve a compromise and reasonable optimization results.

### 3.3. Classifier Adaptation

**Figure 3:**
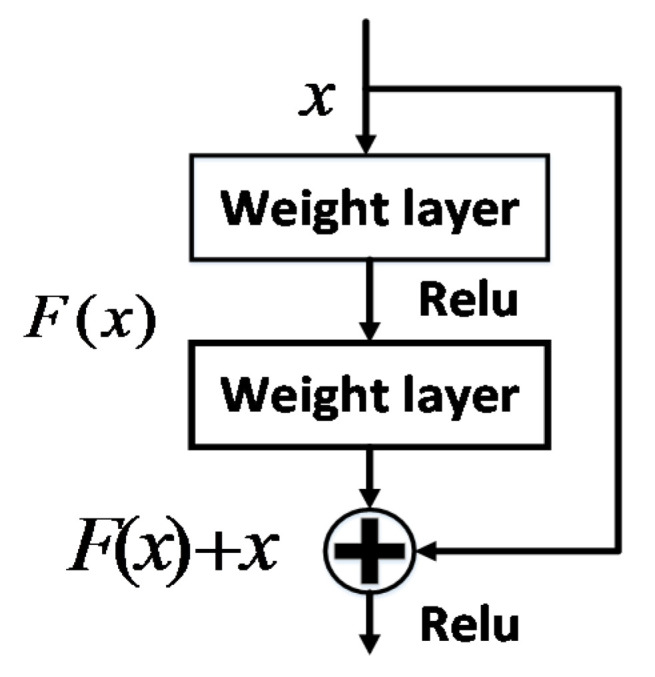
Residual learning: a building block.

In order to reduce the difference of classifiers between two domains, adding classifier adaptation (CA) is also an effective method. CA is motivated by the deep residual learning [2] which is shown in Figure 3 and Domain Adaptation with Residual Transfer Networks [14]. Based on the formula H(x)=F(x)+x in [2], we can deduce our classifier adaptation (CA) function:

(8) $$f_{s}\left(x\right)=\Delta f\left(x\right)+f_{t}\left(x\right)$$

$f_{s}\left(x\right)$ is the output of source classifier and $f_{t}\left(x\right)\;$ is the output of target classifier, ∆f(x)  is a perturbation of classifiers between two domains. Let $x≜f_{t}\left(x\right)$ and $H\left(x\right)=f_{s}\left(x\right)$. There is an assumption that when target domain and source domain are connected, perturbation function ∆f(x)  can be learned from the labeled data in the source domain and the unlabeled data in the target domain. The source label classifier’s loss function can be computed as:

(9) $$\underset{f_{s}\left(x\right)=△f\left(x\right)+f_{t}\left(x\right)}{\min}\frac{1}{n_{s}}\sum_{i=1}^{n_{s}}J\left(\theta_{repr}\left(x_{i}^{s}\right),y_{i}^{s}\right)$$

where *J* is the cross-entropy loss function, and $\theta_{repr}\left(x_{i}^{s}\right)$ denotes the conditional probability that the network attaches label $y_{i}^{s}$ to the sample $x_{i}^{s}$.

Although classifier adaptation (CA) has reduced the difference of source classifier and target classifier, the output of target classifier $f_{t}\left(x\right)$ cannot be guaranteed to fit the target domain very well. Therefore, the entropy minimization principle is used to optimize the parameters and minimize the entropy of conditional distribution of each class in order to encourages the low-density separation among classes of target domain. The target classifier’s loss function can be computed as:

(10) $$\underset{f_{t}}{\min}\frac{1}{n_{t}}\sum_{i=1}^{n_{t}}H\left(f_{t}\left(x_{i}^{t}\right)\right)$$

where *H* is the entropy loss function, $H\left(f_{t}\left(x_{i}^{t}\right)\right)=-\sum_{j=1}^{c}f_{j}^{t}\left(x_{i}^{t}\right)\log f_{j}^{t}\left(x_{i}^{t}\right)$, *c* is the number of classes and $f_{j}^{t}\left(x_{i}^{t}\right)$ denotes the probability that the label is *j* for $x_{i}^{t}$.

### 3.4. Loss Function

According to DAN’s network, this paper uses the classic five convolutional layers and three full connection layers. Each full connection layer *L* learns a nonlinear mapping $h_{i}^{l}=f^{l}\left(W^{l}h_{i}^{l-1}+b^{l}\right)$, where $W^{l}$ and $b^{l}$ are the weights and offsets of the *L*th layer, $h_{i}^{l}$ is the feature representation for $x_{i}$ in the *L*th layer, and $f^{l}$ is the activation function. Let $\theta_{repr}={\left\{W^{l},b^{l}\right\}}_{l=1}^{L}$ represents the parameter set of the convolutional neural networks, the empirical loss function of the whole network is as follows:

(11) $$\underset{\theta_{repr}}{\min}\frac{1}{N}\sum_{i=1}^{N}J\left(\theta_{repr}\left(x_{i}^{t}\right),y_{i}\right)$$

In this paper, we add classifier adaptation (CA) by adding two fully connected layers as residual layers to ensure that $f_{t}\left(x\right)$ does not deviate too far from $f_{s}\left(x\right)$, so Equation (11) is rewritten into Equation (9).

In the standard convolutional neural network, the deep feature representation becomes from generalization to specialization with the layer from low to high. The higher the network layer is, the greater domain’s difference is, which cannot be reduced by fine-tune alone. Therefore, it is necessary to make adaptation on the full connection layers instead of convolutional layers. In this paper, we propose two ways to do domain adaptation on the full connection layers and reduce the difference of domain distribution. One way is to add MK-MMD based multi-layer adaptation, and the other way is to add a domain classification layer $f_{\mathrm{DC}}$. We further extend our method by adding classifier adaptation (CA). Then the three factors are considered into the corresponding loss function, and the loss function of the whole network is as follows:

(12) $$\begin{array}{cc} L= & \underset{f_{s}\left(x\right)=△f\left(x\right)+f_{t}\left(x\right)}{\min}\frac{1}{n_{s}}\sum_{i=1}^{n_{s}}J\left(\theta_{repr}\left(x_{i}^{s}\right),y_{i}^{s}\right)+\lambda \sum_{l=l_{1}}^{l_{2}}d_{k}^{2}\left(D_{s}^{l},D_{t}^{l}\right)\\ & +\gamma \left\{-\sum_{d}1\left[y_{D}=d\right]\log q_{d}-\sum_{d}\frac{1}{D}\log q_{d}\right\}\\ & +\frac{\beta }{n_{t}}\sum_{i=1}^{n_{t}}H\left(f_{t}\left(x_{i}^{t}\right)\right) \end{array}$$

where *λ*, γ and β are the balanced weights of MK-MMD, DC and low-density separation between classes of target domain respectively.

To train a convolutional neural network from scratch requires a large amount of labeled data, which is also impossible for domain adaptation problems, so in this paper the initial network adopts AlexNet model trained on ImageNet 2012 as the pre-trained model. Then we freeze the convolution layer from layer 1 to layer 3 and fine-tunes the 4th and 5th convolution layer, and other parameters are calculated by optimizing the loss function through training.

For different domain adaptation scenarios, the weight of loss function could be different. When we combine MK-MMD, domain confusion and classifier adaptation, we also explore the contribution’s difference with different domain adaptation metric to different scenarios. We also consider the reasonable combination of the two methods. This part is explained in Section 4.

## 4. Experiment Results and Analysis

### 4.1. Data Set 

**Figure 4:**
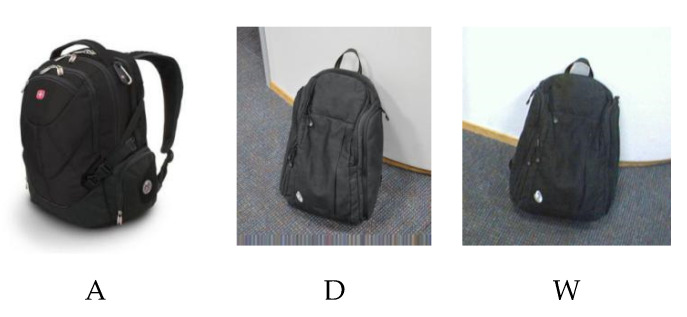
Examples of the Office-31 dataset. (**A**) (Amazon, downloaded from amazon.com), (**D**) (DSLR, captured from digital SLR cameras), (**W**) (Webcam, captured from webcams).

Office-31 [31] is a standard data set in the research field of domain adaptation. The Office-31 dataset contains 31 categories of 4,652 images collected from three separate domains, which are (1) Amazon (A, downloaded from amazon.com) with 2817 images, (2) Webcam (W captured from webcams) with 795 images, and (3) DSLR (D captured from digital SLR cameras) with 498 images. The example images are shown in Figure 4. There are six transfer scenarios in these three domains, which are A –> W, D –> W, W –> D, A –> D, D –> A and W –> A. 

Office-10+Caltech10 dataset contains 10 categories which are selected from Office-31 and Caltech-256. There are four domains in total, three domains (A, W, D) separated from Offce-31 and one domain (C) from Caltech-256. Then six transfer tasks can be built: A –> C, W–> C, D –> C, C –> A, C –> W and C –> D. While Office-10+Caltech has less categories, it will be easier to make adaptation than Office-31 dataset. In this paper, experiments and comparative analysis will be conducted on the two datasets.

In addition to the improved deep adaptation network which combines DC and MK-MMD proposed in this paper, the experiment will be compared with the traditional CNN method, the DAN method only using MK-MMD and the Residual Transfer Networks (RTN) method using classifier adaptation (CA). At the same time, in order to explore the application value and conditions of domain confusion in this network, the experiments of FC6 and FC7 based on DAN and Combination of RTN and DC also will be conducted and compared. Finally, we do the experiments which combining MK-MMD, DC with CA.

### 4.2. Experiment Procedure

In this paper, we use Caffe to develop the proposed deep neural network, which contains the classic five convolutional layers and three full connection layers. Similar to the method of DAN, MK-MMD layers are attached to the three full connection layers to calculate the multi kernel maximum mean discrepancy of feature representation between source domain and target domain in Hilbert space. At the same time, the domain classification layer $f_{\mathrm{DC}}$ (called DC layer), which is derived from the idea of domain confusion, is added after the seventh full connection layer. Besides, two residual layers are added after FC8 to reduce the difference between the source classifier and target classifier. Then the classification loss, domain confusion loss and entropy loss of target classed are also calculated. In order to implement the function of the layer $f_{\mathrm{DC}}$, it is necessary to add the corresponding domain labels 0 and 1 to the data of source and target domains in the model file. And the input data of layer $f_{\mathrm{DC}}$ include the feature representation obtained by the seventh full connection layer and the labels of the two domains. 

Here we use fine-tuning method to train this model. Considering the problem of the amount of data in the training set, we get the parameters of the first three convolutional layers from the pre-training model and freeze them. Then we fine-tune the subsequent convolutional layers and the full connection layers by the way of back-propagation. The random gradient descent method is used in the experiment, which parameter is 0.9. And the learning rate adopts ‘inv’ method, which parameter is 0.75, and the initial learning rate is 0.001. We set the batch size to 64 for all methods, and optimize the learning rate for each model independently. Since all the comparative methods use mmd as test statistic, Gaussian kernel is used to median pairwise squared distances on training data. We perform cross-valuation on labeled source data to select candidate parameters, the weights are selected following the strategy: (1) Try to get the best results by adjusting the mmd weight when we apply the DAN method. Then the mmd weight is fixed, which means we use the same mmd weight in all other methods. (2) Try to get the best results by adjusting the entropy weight when we realize the RTN method and fix the entropy weight. (3) Realize our method C^2^DAN based on the mmd weight and entropy weight. The detail to choose weights is introduced in Section 4.4.

### 4.3. Experiment Results and Analysis

This experiment implements the unsupervised domain adaptation of the improved deep adaptation network C^2^DAN combining DC, CA and MK-MMD, which means DAN+DC+CA, on the Office-31 dataset and Office–10+Caltech-10 dataset. And comparing with the ordinary convolution neural network CNN (Baseline), the classical deep adaptation network DAN [12] and RTN [14], the experimental results are shown in Table 1 and Table 2. 

From the experimental results in Table 1 and Table 2, we can make the following observations: (1) For different domain adaptation scenarios, the domain adaptation method has different effects. For the adaptation between domain D and W, the effect is not obvious because the similarity between the two domains is very high. Then for the adaptation of domain A to D or W, the result is obviously better. (2) Comparing to the DAN and RTN, the average accuracy of the six adaptation scenarios is improved by about 1% with the use of DC. Combining DC with MMD makes the features obtained from source domain more capable of representing the target domain samples, and further reduces the difference of domain distribution. (3) The average accuracy of the method RTN combined with DC is higher than the DAN combined with DC. The main reason is that RTN has classifier adaptation module which can enhance the accuracy of classification. From the data point of view, the use of classifier adaptation can further improve the effect of domain adaptation.

**Figure 5:**
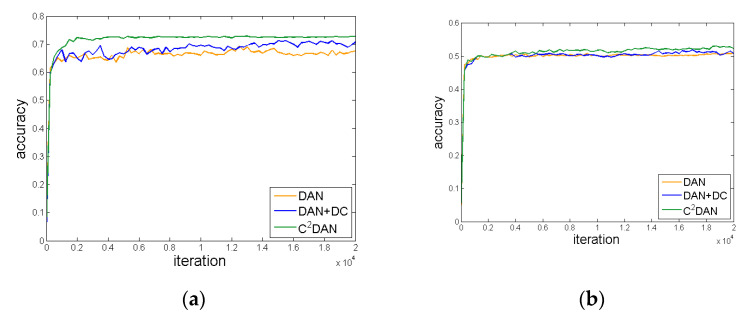
Accuracy of different iterations using different method, (**a**) A-W; (**b**) W-A.

To go deeper into different modules of C^2^DAN, we show the results of each module of C^2^DAN in Table 1 and Table 2. (1) The average accuracy of the method combined with DC6 is not as good as the DAN method using only MK-MMD or the method combining with DC7. Theoretically, with the increase of the number of convolutional layers, the features extracted from each convolutional layer change from generalization to specialization. Therefore, DC7 is selected to make the best domain classifier achieve the maximum domain confusion. (2) Seen from different adaptation scenarios, the adaptation effect of domain A to W is the best. And the comparison of accuracy can be seen in Figure 5a. For the adaptation from domain W to A, the experimental results are not improved obviously, which are shown in Figure 5b. Due to the small amount of data in the source domain W and the poor ability of feature representation for real-world images, the improvement is not obvious when the adapting from the real-world images in domain W to the information-rich website images in domain A. (3) Comparing RTN+DC with C^2^DAN, the average accuracy of C^2^DAN is 73.8% on the Office-31 dataset, which is 0.6% higher than RTN+DC, but the accuracy of C^2^DAN is similar to RTN+DC in Office-10+Caltech-10, which seems little improvement. The difference between the two datasets is Office-10+Caltech-10 has less categories which make it easier to be transferred. Both of these two methods have combined domain confusion with classifier adaptation, the difference is C^2^DAN conducts feature alignment by MK-MMD and RTN by tensor MMD. From the data point of view, the combination of MK-MMD, DC and CA is more effective in more difficult tasks.

### 4.4. Analysis of Weights

In this paper, we explore and analyze the suitable weights of three different domain adaptation loss. The weight parameter of MK-MMD ranges from {0.1, 0.4, 0.7, 1, 1.4, 1.7, 2}, the weight parameter of domain confusion ranges from {0.01, 0.05, 0.1, 0.15, 0.2} and the weight parameter of classifier adaptation ranges from {0.05, 0.1, 0.15, 0.2, 0.25} It’s necessary to select appropriate weights and best combination in different scenarios.

**Figure 6:**
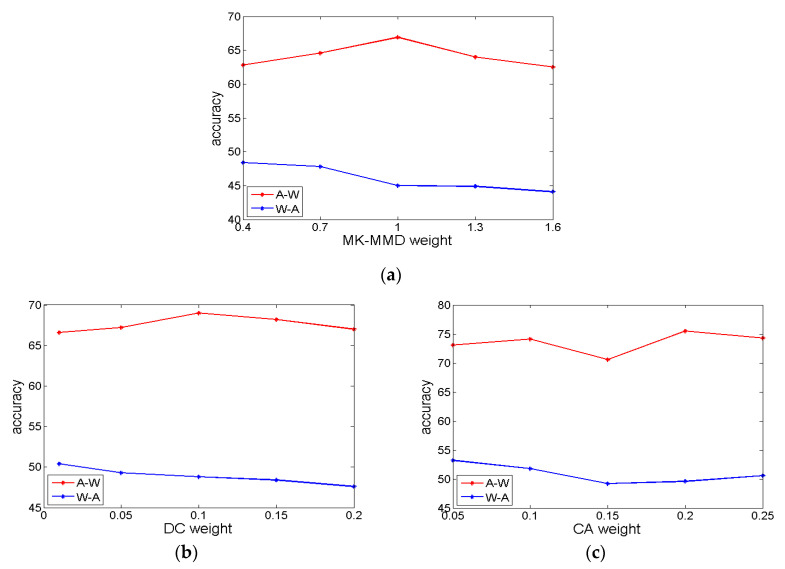
Accuracy comparison in different weights, (**a**)weights of MK-MMD; (**b**) weights of DC; (**c**) weights of CA.

For the weight parameter of MK-MMD, as shown in [12], the adaptation effect of domain A (website images with rich information) to domain D (complex images captured by digital SLR camera) is best when *λ* is 1, as shown in Figure 6a. However, this conclusion is not suitable when the source domain is W or D. From the experimental result, we can see that the effect of domain adaptation becomes worse with the increase of weight and the result is best when *λ* is 0.1. Therefore, in the actual application, the weight of MK-MMD *λ* should be 1 in the case of abundant information in the source domain, such as website images. Then, the weight *λ* should be 0.1 in the case of less information and complex environment in the source domain, such as images captured by web camera or digital SLR camera.

For the weight parameter γ of $f_{\mathrm{DC}}$ layer, we should consider the optimal combination of the two weight parameters. In this paper, we conduct experiments and analyze to get the best fit γ under the optimal *λ* for every adaptation scenario. When the source domain is A and *λ* is 1, the best result can be obtained if γ equals 0.1. The comparison of different weights γ from domain A to domain W is shown in Figure 6b. Meanwhile, when the source domain is W or D and *λ* is 0.1, the best result can be obtained if γ equals 0.01. The comparison of different weights γ from domain W to domain A is shown in Figure 6b.

For the weight parameter β of entropy loss, we should consider the optimal combination of the three weight parameters. In this paper, we conduct experiments and analyze to get the best fit β under the optimal *λ* and γ for every adaptation scenario. When the source domain is A and *λ* is 1 and γ equals 0.1, the best result can be obtained if β equals 0.2. The comparison of different weights β from domain A to domain W is shown in Figure 6c. Meanwhile, when the source domain is W or D and *λ* is 0.1 and γ is 0.01, the best result can be obtained if β equals 0.05. The comparison of different weights β from domain W to domain A is shown in Figure 6c.

From the experimental results, as a domain adaptation method, the weight of domain confusion loss should be one tenth of MK-MMD loss. In theory, domain confusion aims to make the most distinguishable feature representation get the worst classification effect. If the weight of $f_{\mathrm{DC}}$ is too high, the training will tend to domain confusion at the beginning, in which case, feature representation with rich and effective information cannot be learned. Therefore, the weight of domain confusion should be relatively small. At the same time, the fundamental purpose of domain adaptation is to obtain domain invariant feature representation. From this point of view, MK-MMD narrows the distribution difference in the mapping space by narrowing the distance of feature representation to obtain domain invariant feature, but domain confusion only judges the degree of domain confusion and improves the domain invariance of feature representation by optimizing its loss. It can be said that MK-MMD acquire domain invariance actively, but domain confusion is a passive verification. MK-MMD plays a stronger role and domain confusion further improve the effect of domain adaptation at this level. As a result, the proportion of domain confusion loss to the total loss function is less than that of MK-MMD loss. 

In summary, when domain adaptation is carried out from the domain with rich obvious information (e.g. network images) to the domain with less complex information (e.g. real word camera images), the weight of MK-MMD is 1, the weight of domain confusion is 0.1 and the weight of classifier adaptation is 0.2. For reverse domain adaptation scenario, the weight of MK-MMD is 0.1, the weight of domain confusion is 0.01 and the weight of classifier adaptation is 0.05. The weights above make the combination of MK-MMD, domain confusion and classifier adaptation more reasonable and lead to better result.

## 5. Application on Vehicle Classification

### 5.1. The Introduction of the Dataset

This paper uses Comprehensive Cars (CompCars) [32] dataset to carry out unsupervised domain adaptation vehicle classification experiments. CompCars dataset is one of the largest datasets for fine-grained vehicle recognition. This dataset consists of two parts, vehicle image dataset from website (data) and vehicle image dataset from road surveillance (sv_data).

**Figure 7:**
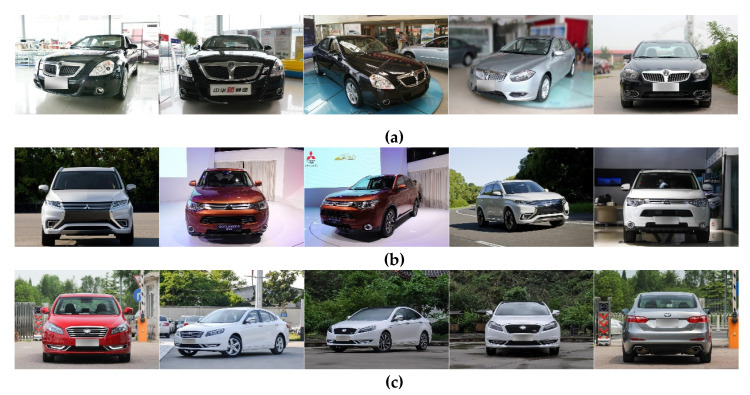
website vehicle images, (**a**) Zhonghua, (**b**) Mitsubishi, (**c**) Besturn

Each section contains 163 types of vehicles, each of which is subdivided into smaller categories according to specific models and years. The model of the experiment is based on AlexNet. The model level is not deep enough and the capability is limited. Therefore, 20 kinds of vehicles are selected for unsupervised domain adaptation experiments, instead of using the whole dataset. The examples of website vehicle image dataset in CompCars are shown in Figure 7.

From the above images, we can see that the shooting angle of the vehicle in the website dataset is not the same. The front, side and back angles are all included. But in the surveillance images, the vehicles are taken from the front with only slight angle change. The angle of view of side and back has changed too much and the distribution is totally different, so this kind of images is not suitable for domain adaptation training. Therefore, we need to filter the website images and only select the pictures taken from the front and the side view which include the vehicle head information.

**Figure 8:**
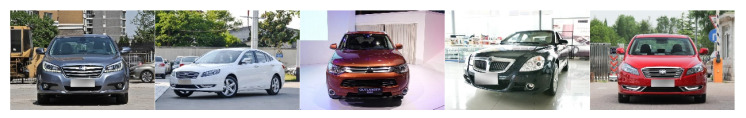
filtered website vehicle images.

**Figure 9:**
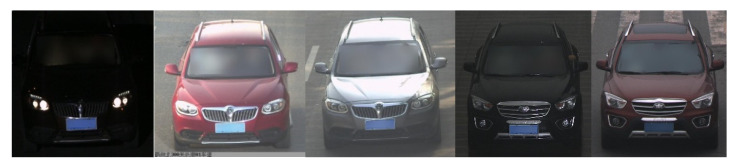
Examples of sv_data.

The examples are shown in Figure 8. The examples of the surveillance vehicle dataset sv_data are shown in Figure 9.

From Figure 9, we can see that the surveillance pictures are taken from the front angle but under different weather and illumination conditions. In this experiment, the selected website images are used as labeled source domain and the surveillance vehicle images belong to unlabeled target domain. Although the website images are taken approximate frontally, they are collected under good lighting conditions and the angle is biased, which results in the distribution differences between the source domain and the target domain due to the angle of view, illumination and other factors. The vehicle classification experiments using these two parts of data can prove the validity of the model in solving the problem of domain shift.

Details of the dataset used in this experiment are as follows. The website dataset(data) contains 20 categories, totaling 6425 pictures, and the surveillance dataset(sv_data) contains 20 categories, totaling 6960 pictures. The categories and their quantities are shown in the Table 3 below.

### 5.2. Experiments Details and the Result

We use four methods to conduct comparative experiments: convolutional neural network CNN (baseline), DAN, the proposed deep adaptation network combining domain confusion with MK-MMD and the combination of DC, CA and MK-MMD. According to the analysis result, the experiment adapts from the domain with rich information network images to the domain with surveillance images including less rich information. Therefore, in our experiment, the loss weight of MK-MMD is 1, the loss weight of domain confusion is 0.1, and the loss weight of classifier adaptation is 0.1.

The experiment is carried out under the framework of caffe. We need to add MK-MMD layer, domain confusion layer and residual layers, and then recompile caffe. Twenty types of vehicles are labeled with "0" ~ "19". During the training process, there is no label information in the vehicle image of the target domain. The accuracy results of vehicle classification experiments are shown in Table 4.

First of all, we need to point out that although the CompCars dataset is used for fine-grained classification, the purpose of our experiment is to verify the ability of unsupervised domain adaptation method to solve the model degradation problem when the source domain training model is applied in the target domain (without any labels). The emphasis is on the improvement of the domain adaptation effect, so it is inappropriate to compare with advanced fine-grained classification model.

Meanwhile, it can be seen from the examples of website images and surveillance images that the source and target images used in this experiment have various changes in background, illumination, angle of view, etc. The domain shift is large, so the overall accuracy of this experiment is not high. However, even in this complex case, the method in this paper has achieved better results. In the follow-up study, assuming that the available source and target domain data are purer or more task-specific, the domain adaptation effect of this method will be better. The experimental results are analyzed as below.

From the experimental results, it can be seen that the accuracy of unsupervised domain adaptation in vehicle classification task from the labeled source domain to the unlabeled target domain is 35.1% without any domain adaptation components. The DAN method based on the MK-MMD component has a classification accuracy of 44.9%, which is 9.8% higher than that of baseline. This proves that the domain adaptation can overcome inter-domain distribution differences caused by the variation of illumination and angle of view. The classification accuracy of DAN+DC and RTN+DC are both higher than the original methods DAN and RTN, which can prove the effectiveness of DC. The accuracy of our method C^2^DAN is 50.7%, 5.8% higher than DAN and 6.4% higher than RTN. It can be in inferred that the classification accuracy is greatly enhanced because the huge difference between source domain and target domain has been reduced by CA and DC. The results demonstrate that the combination of MK-MMD, DC and CA is reasonable and efficient. Moreover, comparing with the standard domain adaptation datasets Office-31 and Office-10+Caltech-10, the improvement of C^2^DAN on CompCars is more significant, which means C^2^DAN has powerful adaptation ability on challenging tasks. 

### 5.3. Accuracy and Analysis of Various Categories

Here, we compare and analyze the classification accuracy of each vehicle type. The accuracy results of each vehicle type are shown in the following Table 5.

From the results above, the following conclusions can be drawn:

(1) It can be seen that the classification accuracy of 17 types of vehicles has been improved after using the proposed DAN+DC or C^2^DAN methods. Compared with that of the DAN method, only one type has decreased, the data in the table show that the difference is little and the decline is not serious. The accuracy of 85% vehicle types have been improved, which proves that the proposed method is reasonable and effective.
(2) For vehicle types with less data, such as the Besturn and Dongfengfengdu cars, in which type the number of samples in source domain and target domain are both less than 2% of the total number of datasets, the proposed method improves the accuracy of vehicle classification by 28.5% and 5.5% respectively compared with the DAN method, and improves by 38.7% and 13.1% compared with the method using only CNN. It proves that the proposed method’s superiority is obvious. The feature extracted by the model in a limited number of samples greatly improves the representation ability in the target domain. By enhancing the feature invariance, the distribution difference between the source domain and the target domain is further reduced.
(3) For the performance degradation of some classes, the main reason is that MK-MMD is an active acquisition while domain confusion is a passive verification for domain invariant feature. Besides, when the domain invariant property of the feature extracted by MK-MMD has reached the optimum level, the improvable space is very limited and the balance training of two kinds of loss may sacrifice the ability of domain adaptation in some categories. However, the sacrifice degree of this part is not too large. It is within the acceptable range and the accuracy of most categories has been improved.

Generally speaking, the proposed method indeed improves the effect of domain adaptation in the whole vehicle classification dataset.

## 6. Conclusions

This paper proposes an improved deep adaptive network C^2^DAN, which combines MK-MMD with Domain Confusion (DC) and Classifier Adaptation (CA). DC is added to learn domain invariant features by using a domain classification layer to perform adversarial training. We add residual blocks in source classification layer to preform CA which can reduce the discrepancy between source classifier and target classifier.

Experiments on standard domain adaptation datasets Office-31, Office-10+Caltech-10 and vehicle classification dataset CompCars show that: (1) DC and CA are both efficient to reduce the domain shift, which means the proposed method can improve the adaptation ability when knowledges are transferred form labeled source domain to unlabeled target domain. (2) The most suitable weights of MK-MMD and the best weight combination of MK-MMD, DC and CA in different transfer scenarios are explored from theory and experiments. (3) When used in challenging transfer tasks, C^2^DAN shows great advantages to reduce the huge differences between two domains. It can be inferred that DC is helpful to align the feature distribution with the use of MK-MMD. CA is also necessary especially in difficult transfer scenarios, we can’t use shared classifier to get good performance because the classification standards of source domain and target domain could be quite different.

In the future work, domain adaptation is going to face challenges that are harder than ever, DC and CA are both illuminating ideas which can be added to new domain adaptation methods. Furthermore, our work is expected to be applied in other computer vision tasks such as fine-grained classification and object detection.

## Figures and Tables

**Table 1:** Result of unsupervised domain adaptation experiment on Office-31 dataset.

	A-W	D-W	W-D	A-D	D-A	W-A	Average
Baseline	60.6(~0.6)	95.0(~0.5)	99.1(~0.2)	59.0(~0.7)	49.7(~0.3)	46.2(~0.5)	68.2
DAN [12] RTN [14]	66.9(~0.6) 70.0(~0.4)	96.3(~0.4) 96.8(~0.2)	99.3(~0.2) 99.6(~0.1)	66.3(~0.5) 69.8(~0.2)	52.2(~0.3) 50.2(~0.4)	49.4(~0.4) 50.0(~0.6)	71.6 72.7
DAN+DC (fc6)	67.3(~0.6)	96.0(~0.3)	99.1(~0.2)	66.0(~0.7	51.5(~0.3)	49.6(~0.5)	71.5
DAN+DC (fc7) RTN+DC C^2^DAN	69.0(~0.7) 73.0(~0.7) 74.0(~0.6)	96.2(~0.4) 97.3(~0.5) 96.6(~0.7)	99.5(~0.2) 99.6(~0.1) 99.6(~0.1)	67.0(~0.6) 70.8(~0.2) 71.5(~0.3)	52.5(~0.5) 50.4(~0.4) 53.0(~0.6)	50.2(~0.5) 51.8(~0.6) 52.2(~0.4)	72.5 73.8 74.4

**Table 2:** Result of unsupervised domain adaptation experiment on Office-10+Caltech10 dataset.

	A-C	W-C	D-C	C-A	C-W	C-D	Average
Baseline	82.6(~0.3)	75.8(~0.3)	77.1(~0.5)	90.5(0.1)	79.6(0.2)	83.5(0.5)	81.5
DAN [12] RTN [14]	86.0(~0.5) 88.1(~0.2)	81.5(~0.2) 85.6(~0.1)	81.8(~0.3) 84.1(~0.2)	92.0(~0.5) 93.0(~0.1)	90.6(~0.5) 96.3(~0.3)	90.2(~0.3) 94.2(~0.2)	87.0 90.2
DAN+DC (fc6)	85.0(~0.1)	80.4(~0.3)	80.0(~0.3)	91.7(~0.3)	85.6(~0.2)	88.6(~0.2)	85.2
DAN+DC (fc7) RTN+DC C^2^DAN	86.4(~0.2) 88.4(~0.4) 88.7(~0.3)	82.2(~0.5) 86.5(~0.2) 86.3(~0.5)	82.5(~0.1) 85.3(~0.3) 85.0(~0.5)	92.8(~0.3) 93.7(~0.3) 93.5(~0.2)	92.3(~0.5) 96.3(~0.1) 97.0(~0.3)	91.3(~0.5) 95.0(~0.2) 95.6(~0.1)	87.9 90.8 91.0

**Table 3:** The categories and quantities.

	**Acura**	**Benz**	**Besturn**	**BYD**	**Changan**
data	157	570	72	356	405
sv_data	370	155	68	395	465
	Dongfengfengdu	Geely	Haima	Honda	Hyundai
data	46	426	69	360	645
sv_data	92	576	203	380	572
	Jeep	Lexus	MAZDA	Mitsubishi	Nissan
data	200	283	314	275	431
sv_data	304	188	371	281	462
	Shuanglong	Toyota	Volkswagen	Volvo	Zhonghua
data	190	511	553	370	193
sv_data	264	572	533	598	111

**Table 4:** Comparison of vehicle classification accuracy.

Method	Accuracy
CNN (Baseline)	0.351
DAN [12]	0.449
RTN [14]	0.443
DAN+DC	0.476
RTN+DC	0.456
C^2^DAN (DAN+DC+CA)	0.507

**Table 5:** Comparison of classification accuracy for 20 vehicle types.

	CNN (Baseline)	DAN	DAN + DC	C^2^DAN
Acura	0.511	0.600	0.614	0.608
Benz	0.265	0.696	0.587	0.781
Besturn	0.118	0.220	0.505	0.206
BYD	0.083	0.387	0.332	0.504
Changan	0.606	0.326	0.328	0.338
Dongfengfengdu	0.054	0.130	0.185	0.054
Geely	0.474	0.534	0.520	0.641
Haima	0.000	0.039	0.060	0.014
Honda	0.431	0.281	0.389	0.409
Hyundai	0.271	0.470	0.472	0.530
Jeep	0.740	0.815	0.803	0.869
Lexus	0.617	0.399	0.479	0.724
MAZDA	0.218	0.498	0.496	0.517
Mitsubishi	0.238	0.476	0.605	0.514
Nissan	0.530	0.510	0.574	0.500
Shuanglong	0.273	0.401	0.409	0.391
Toyota	0.196	0.222	0.234	0.195
Volkswagen	0.580	0.656	0.658	0.714
Volvo	0.582	0.698	0.652	0.679
Zhonghua	0.234	0.612	0.622	0.712
